# Domains of life satisfaction and perceived health and incidence of chronic illnesses and hospitalization: evidence from a large population-based Chinese cohort

**DOI:** 10.1186/s12889-022-14119-3

**Published:** 2022-09-08

**Authors:** Kaiwen Bi, Shuquan Chen, Paul S. F. Yip, Pei Sun

**Affiliations:** 1 Department of Social Work and Social Administration, University of Hong Kong, Pok Fu Lam, Hong Kong, China; 2 Department of Psychology, School of Social Science, Tsinghua University, Beijing, China; 3 Department of Counseling and Clinical Psychology, Teachers College, Columbia University, New York, USA; 4 Hong Kong Jockey Club Center for Suicide Research and Prevention, University of Hong Kong, Hong Kong, China

**Keywords:** Longitudinal, Life satisfaction, Physical health, Chronic health condition, Hospitalization

## Abstract

**Background:**

Global life satisfaction has been consistently linked to physical health. A deeper and culturally nuanced understanding of which domains of satisfaction may be responsible for this association has implications for developing novel, scalable, and targeted interventions to improve physical health at the population level.

**Objectives:**

This cohort study draws participants from the China Family Panel Studies (CPFS), a nationally representative cohort of 10,044 Chinese adults to assess the *independent* associations between three important domains of life satisfaction (and their changes) and indicators of physical health.

**Results:**

A total of 10,044 participants were included in the primary analysis (4,475 female [44.6%]; mean [SD] age, 46.2 [12.1] years). Higher baseline levels of satisfaction with job, marriage, and medical services were independently associated with better perceived physical health (0.04 < β values < 0.12). Above and beyond their baseline levels, increases in satisfaction with job, marriage, and medical services were independently associated with better perceived physical health (0.04 < β values < 0.13). On the contrary, only higher baseline levels of and increases in satisfaction with marriage showed prospective associations with lower odds of incidence of chronic health condition and hospitalization (0.84 < ORs < 0.91).

**Conclusions:**

These findings provide policymakers and interventionists interested in leveraging psychological health assets with rich information to rank variables and develop novel interventions aimed at improving wellbeing at the population level.

**Supplementary Information:**

The online version contains supplementary material available at 10.1186/s12889-022-14119-3.

## Introduction

Potentially modifiable health assets such as purpose in life and optimism may exert influence on health outcomes and health behaviors, even after accounting for a comprehensive range of confounders including personality traits and baseline health status [1–3]. Targeting these assets at the population level may hold promise to reduce the cost of healthcare [1]. As a promising health asset, life satisfaction has shown robust associations with health outcomes (e.g., lower mortality, lower risk of hospitalization and chronic diseases) and health behaviors (e.g., not smoking, physical exercise, and fat intake restriction) [4–7]. Previous research suggests that life satisfaction may exert its influence on health through multiple pathways, both directly via physiological processes such as reducing inflammation and indirectly via activating adaptive health behaviors and buffering stress [8–10]. Furthermore, accumulating evidence reveals the potentially modifiable nature of life satisfaction [11, 12].

Taken together, these important findings suggest that improving one’s life satisfaction may be a viable path to improving physical health at the individual level and reducing chronic disease, hospital stays, and thereby health care costs at the societal level. Many existing studies were, however, limited by study design [6, 13] (e.g., less generalizable/western samples, cross-sectional design) and/or by their reliance on global life satisfaction without considering various domains of life satisfaction such as satisfaction with job and family life [4–6]. Even among studies that focused on one specific domain (e.g., marriage, job, etc.), many still adopted a cross-sectional design, examined western samples, and/or focused on one health outcome [13, 14]. Additionally, most previous studies focused on life satisfaction’s accumulated lifetime effects on health without evaluating whether changes in life satisfaction over time are independent predictors of health [4–6, 15]. Due to these limitations, not enough is known about (1) which specific domains of life satisfaction may be the drivers of the observed association, especially in collectivistic countries, and (2) whether short-term increases in domains of life satisfaction may be associated with physical health at follow-up, independent of baseline levels.

Recently, several critical studies have extended this line of research in important ways. Willroth et al. adopted a longitudinal design and found that being happy and becoming happier predict better physical health and lower mortality in the United States and Japan [16]. Nakamura et al. assessed the association between physical health and changes in seven life satisfaction domains including home, city/town, family, financial situation, income, daily life, and leisure among a U.S. cohort [17]. These authors identified differential associations between domains of life satisfaction and indicators of physical health. First, all domains were associated with better self-reported health. Second, whereas increases in satisfaction with financial situation and health predicted fewer total chronic health conditions, increases in satisfaction with health additionally predicted lower mortality at follow-up [17]. Taken together, these more recent findings suggest that both overall life satisfaction and some specific domains of life satisfaction (especially those related to health and economics) may be drivers of better physical health. However, still not enough is known about whether other unexamined domains of life satisfaction that are overlapping, but still distinct, vs. those examined previously such as satisfaction with marriage, job, and medical services could also predict better physical health, independent of other domains, especially in non-western samples [18].

In the present study, we aimed to build on previous studies by analyzing a large nationally representative Chinese longitudinal sample that assessed three important domains of life satisfaction including job, marriage, and medical services. Specifically, we examined the *independent* associations between three indicators of physical health and a total of six longitudinal predictors including both the *levels* and *changes* in these domains of life satisfaction.

## Methods

### Study population

**Fig. 1:**
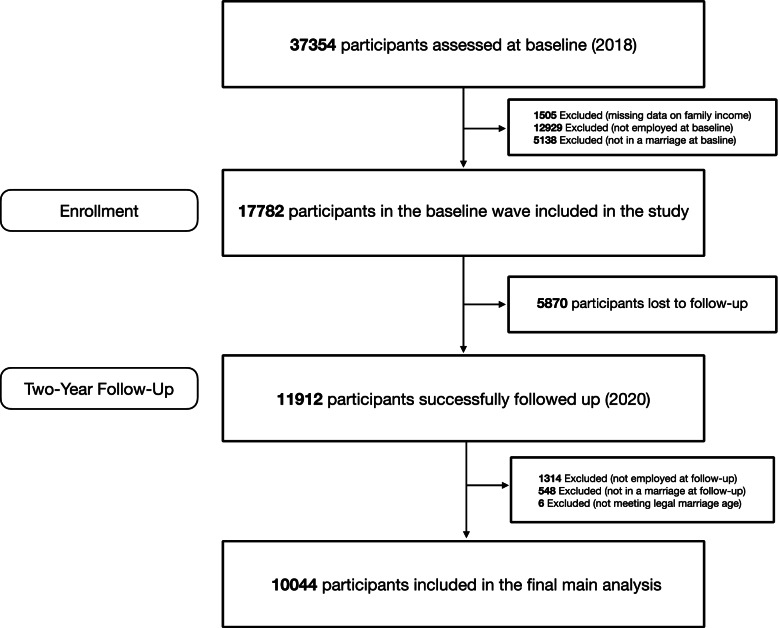
Participant flow chart

Data were from the China Family Panel Studies (CFPS). CFPS is an ongoing project that follows a nationally representative cohort of Chinese families biannually. In 2010, the year CFPS was launched, 14,960 families from 25 province-level regions in China were assessed [19]. Six waves of data (2010, 2012, 2014, 2016, 2018, and 2020) were available. In the present study, we used wave 2018 and wave 2020 to examine the associations of three domains of life satisfaction (job, marriage, and medical services) and health. We chose these two waves for two reasons: (1) in earlier waves, some domains of satisfaction were not assessed; and (2) the full version of the scale measuring job satisfaction was only present in the last two waves (for a detailed descriptions on how and whether these domains were assessed in each wave, see Table S1). In total, CFPS assessed 37,354 participants in 2018. We have only included participants who were employed and married in both waves to simultaneously examine the independent contributions of domains of satisfaction including job, marriage, and medical services. Specifically, we excluded 1505 participants due to missing data on family income and 18,067 participants because they were not employed and/or married in the baseline wave, resulting in 17,782 participants in the baseline wave. 5,870 participants were lost to follow-up, which resulted in 11,912 participants at follow-up. Chi-squared tests revealed that compared to those who were followed up, those who were not were less educated and less likely to possess party membership; no significant differences were found in other demographics including age, sex, residential possession, and family income. We then further excluded 1,862 participants who were not employed and/or married at follow-up, leaving 10,050 participants. Finally, two females and four males whose age in the baseline wave was below the marital age limits in China (20 and 22, respectively) were excluded. After all exclusion procedures, a total of 10,044 participants were retained at baseline in the final main analysis (for detailed participant flow chart, see Fig. 1). All results were maintained when these six participants were included. As CFPS is publicly available, the study was exempt from review by the institutional review board of Tsinghua University.

## Measures

### Satisfaction with job

Satisfaction with job was assessed using a six-item scale measuring one’s satisfaction with current income, job security, working environment, working time, promotion opportunity, and general perception on a five-point Likert scale from 1 (*very unsatisfied*) to 5 (*very satisfied*). The item measuring satisfaction with job opportunity was excluded because of a large amount of missingness at all waves vs other items (e.g., 65.4% vs. 21.8% for general satisfaction with job in 2018). An example item is “In general, how satisfied are you with this job?”. Item scores were averaged with higher scores indicating greater satisfaction with job. The internal consistency of the scale is good (Cronbach αs > 0.82).

### Satisfaction with marriage

Satisfaction with marriage was assessed using three items (“In general, are you satisfied with your current marriage/cohabitation?”, “Are you satisfied with the economic contribution that your spouse/partner makes to the family?”, and “Are you satisfied with the contribution on housework that your spouse/partner makes to the family?”) on a five-point Likert scale from 1 (*very unsatisfied*) to 5 (*very satisfied*). Item scores were averaged with higher scores indicating greater satisfaction with marriage. The internal consistency of the scale is acceptable (Cronbach αs >  = 0.76).

### Satisfaction with medical services

Satisfaction with medical services was assessed using one item (“Are you satisfied with the overall medical service?”) on a five-point Likert scale from 1 (*very unsatisfied*) to 5 (*very satisfied*).

### Covariates

In all models, we adjusted for a wide range of covariates. Categorical covariates in the present study included baseline sex, education level, residential possession or hukou [20], and Chinese Communist Party membership, with female (vs. male), high school or below (vs. some college or above), rural residents (vs. urban residents) and non-party member (vs. party member) as the reference group respectively. Continuous covariates included age and log-transformed family income [21]. We included age, sex, education, residential possession, and family income because they had been linked to health and were commonly controlled for in the literature [17, 22]. We included Chinese Communist Party membership as a covariate because it being a nationally integrated political party has the founding and dominant status in China, and its membership may confer better access to resources. The membership has been conceptualized as an indicator of social capital linked to higher health care utilization [23]. Furthermore, perceived physical health at baseline was additionally controlled for in the multivariable linear regression models predicting perceived physical health at follow-up.

### Outcomes

#### Perceived physical health

Perceived physical health was assessed with the question, “How would you rate your health status?” on a five-point Likert scale from 1 (*excellent*) to 5 (*poor*). Item scores were reverse scored so that higher scores indicate better health.

#### Chronic health condition onset

Chronic health condition onset was measured with the question, “During the past six months, have you had any doctor-diagnosed chronic disease?”. Possible answers were “Yes” and “No”.

#### Hospitalization

Hospitalization was measured with the question, “In the past year, were you ever been hospitalized due to illness?”. Possible answers were “Yes” and “No”.

#### Statistical analysis

All analyses were conducted in R version 4.1.3 (R Project for Statistical Computing) via base R functions and *car*, *psych*, *forestplot*, *bruceR*, *mice*, and *miceadds* packages [24–30].

To examine if increases in domains of life satisfaction predict better physical health and lower odds of chronic condition onset and hospitalization above and beyond their baseline levels, we calculated the difference of participants’ scores on each domain’s life satisfaction at baseline and at follow-up such that a positive score indicates that a participant’ satisfaction has increased over time. These values, along with their baseline levels, were then used as predictors of physical health in (logistic) regression models. Levels of multicollinearity were low across all models (variance inflation factors < 2.5).

In total, we built one regression model with perceived physical health (continuous) as the outcome and two binary logistic regression models with chronic condition onset (binary) and hospitalization (binary) as the outcomes, respectively. In each of the models, we entered both changes and levels of three domains of life satisfaction (job, marriage, and medical services) simultaneously while controlling for covariates including age, sex, education level, residence possession, log-transformed family income, and membership of Chinese Community Party. To predict chronic health condition onset and hospitalization, in each logistic regression model, we removed participants who reported chronic health conditions and hospitalization in the baseline wave, respectively. Doing so resulted in 8542 and 8975 cases in these two models, respectively. Lastly, because some individuals were nested within families, clustered robust standard error was calculated in all regression models at the family level using family identifiers via the (g)lm.cluster () functions of the *miceadds* package [30]. All continuous predictors were standardized (mean [*M*] = 0, standard deviation [*SD*] = 1) to facilitate effect size comparison and interpretation of study findings.

### Sensitivity analyses

To evaluate the robustness of the findings, we conducted the following sensitivity analyses: (1) reanalysis of all models while additionally adjusting for Big-Five personality traits assessed by a validated brief Chinese version in 2018 [31]; (2) reanalysis of all models using only the items measuring general satisfaction with job and marriage in addition to the single-item question measuring satisfaction with medical services (i.e., three single-item questions corresponding to each domain);(3) reanalysis of all models using 10 imputed datasets by chained equations with binary variables imputed using logistic regression and continuous variables imputed using predictive mean matching via the mice() function of the *mice* package; and (4) reanalysis of all models using the alternative baseline CFPS wave (2014) and two different observation periods (i.e., from 2014 to 2018 and from 2014 to 2020).

## Results

At baseline, participant age ranged from 20 to 84 (mean [SD] age, 46.2 [12.1] years; 4475 females [44.6%]; Table 1). The vast majority of participants (92.6%) were of the Han ethnic group (China’s majority ethnic group), while 7.4% were ethnic minorities. With regards to population-level changes in the domains of life satisfaction between the timepoints, separate paired t-tests revealed that while satisfaction with marriage declined slightly over the two-year observation period, *p* < 0.001, Cohen’s *d* = -0.07, both satisfaction with job and with medical services improved slightly, *p.s.* < 0.001, Cohen’s *d.s.* = 0.14 and 0.16, respectively.

**Table 1:** Participant baseline characteristics

Participant baseline characteristics	*M* (*SD*) or N (%)
Age in 2018 (years)	46.2 (12.1)
Sex	
Female	4,475/10,044 (44.6)
Male	5,569/10,044 (55.5)
Education in 2018	
High school or below	8,690/10,044 (86.5)
Some college or above	1,353/10,044 (13.5)
Residence possession in 2018	
Rural	7,915/10,035 (78.9)
Urban	2,120/10 035 (21.1)
Family income in 2018	7,1614.7 (78,109.5)
Party membership in 2016	
Non-member	9,051/10,044 (90.1)
Member	993/10,044 (9.9)

*Note:* Descriptive statistics were not imputed. All variables at baseline were controlled for in all statistical analyses. Percentages of sex do not add up to 100% because of rounding

We report standardized regression coefficients, *t* statistics, and 95% confidence intervals (CIs) from multivariable linear regressions predicting perceived physical health in Table 2. The multivariable linear regression with perceived physical health as the outcome revealed that all domains’ baseline levels were unique predictors of perceived physical health at follow-up, after adjustment of covariates including age, sex, education, family income, residential possession, and party membership (Table 2). Among them, job satisfaction level (i.e., being satisfied with one’s job in the baseline wave) shows the largest effect size with the highest standardized beta coefficient. Above and beyond domain levels, positive changes of satisfaction with job, marriage, and medical services were also robust predictors of better perceived physical health at follow-up (Table 2). Among them, job satisfaction change shows the largest effect size with the highest standardized beta coefficient.

**Table 2:**
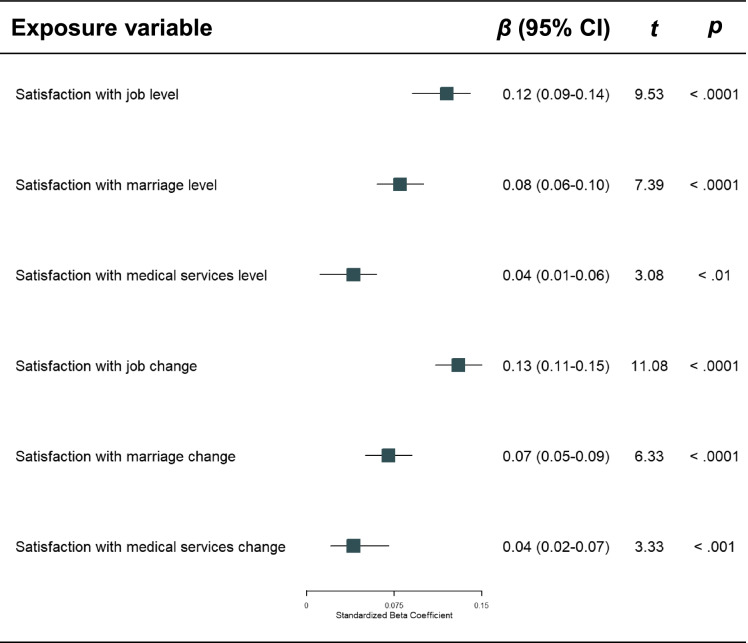
Multivariable linear regression model predicting perceived physical health adjusted for covariates

*Note:* Covariates controlled for included sex, age, education level, residential possession (rural vs. urban), family income, party membership, and perceived physical health in the baseline wave

*CI* Confidence interval

After excluding those who reported chronic health conditions at baseline, 8542 participants were left. Among them, 828 participants (9.8%) experienced at least one chronic health condition at follow-up. In the logistic regression predicting objective doctor-diagnosed chronic health condition onset at follow-up, the only domain level predictor was the level of satisfaction with marriage (Table 3). Above and beyond domain levels, only positive changes in one’s satisfaction with marriage predicted lower odds of chronic health condition onset (Table 3).

**Table 3:**
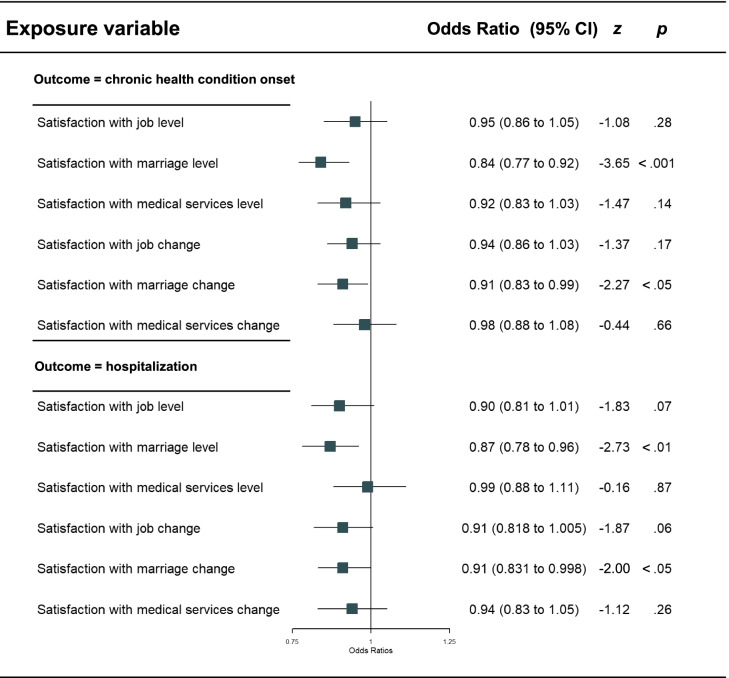
Multivariable logistic regression models predicting chronic health condition onset and hospitalization adjusted for covariates

*Note:* Covariates controlled for included sex, age, education level, residential possession (rural vs. urban), family income, and party membership

*CI* Confidence interval

After excluding those who reported hospitalization at baseline, 8975 participants were left. Among them, 623 participants (7.0%) experienced hospitalization at follow-up. In the logistic regression predicting hospitalization, the only domain level predictor we found was the level of satisfaction with marriage (Table 3). Above and beyond domain levels, only positive changes in satisfaction with marriage predicted lower odds of hospitalization (Table 3).

### Results of sensitivity analyses

Across the first three sensitivity analyses (i.e., additional adjustment of Big-Five personality traits, use of three single-item questions, and analysis of multiply imputed datasets), baseline satisfaction with job, marriage, and medical services were unique predictors of perceived physical health. In addition, across all these additional analyses, baseline levels of satisfaction with marriage and their positive changes were unique predictors of lower odds of chronic health condition onset and baseline levels of satisfaction with marriage predicted lower odds of hospitalization (Tables S2-10). The analysis of Wave 2014 and 2020 revealed that (1) baseline level of and change in satisfaction with job, marriage, and medical services were unique predictors of perceived physical health, (2) baseline level of and increases in satisfaction with marital satisfaction were unique predictors of lower odds of chronic health condition onset, and (3) increases in satisfaction with marriage predicted lower odds of hospitalization (Tables S11-13). The analysis of Wave 2014 and 2018 revealed that (1) baseline level of and change in satisfaction with job, marriage, and medical services were unique predictors of perceived physical health and that (2) baseline levels of satisfaction with marriage predicted lower odds of hospitalization (Tables S14-16).

## Discussion

The study shows that both baseline and change of three domains of life satisfaction are independently associated with better physical health as operationalized by perceived physical health, while satisfaction with marriage exhibits association with all three indicators of health. That all domains’ increases were associated with better perceived physical health suggests that similar gains in physical health may be observed if interventions aimed at improving satisfaction with marriage, job, and medical services are deployed at scale. Among them, satisfaction with marriage might be the most valuable target to be incorporated into novel intervention efforts aimed at improving physical health, given its robust association with not only better perceived physical health, but also lower odds of chronic health condition onset and hospitalization across main analyses and sensitivity checks with one exception (Table S16).

By entering all predictors of domains of life satisfaction into the models, we found that baseline and increase in satisfaction with one’s job, marriage, and medical services predicted better perceived physical health. These associations remained not only in the main analyses but also across all sensitivity checks including stricter adjustment of covariates, multiple imputations, and the use of alternative waves and scale versions, therefore highlighting the robustness of our findings. These findings were consistent with findings from a U.S. sample in that higher satisfaction with all examined domains of life satisfaction was found to be prospectively associated with better self-reported physical health [17]. However, somewhat inconsistent with their finding that higher satisfaction with financial situation, but not family life, predicted fewer chronic health conditions, we found that satisfaction with marriage, a construct closely related to family life, to be a predictor of chronic health condition onset, which might result from our participants coming from a non-western collectivistic culture (China), where family harmony was more valued vs. individualistic countries [32] and/or methodological differences. Finally, our findings were also consistent with a longitudinal study that found that greater overall life satisfaction predicted better physical health, albeit unsurprisingly of lower magnitude [16].

By contrast, while satisfaction with job shows the largest associations (i.e., largest standardized coefficients) with perceived physical health, we found that satisfaction with marriage was the only domain that predicted lower odds of chronic condition health onset and hospitalization. However, in some sensitivity analyses, satisfaction with job was associated with lower odds of chronic health condition onset or hospitalization (Tables S4-12). Future studies are needed to further clarify whether satisfaction with job could consistently predict chronic health condition onset and hospitalization in the Chinese context. The greater contribution of satisfaction with marriage to lower odds of chronic health onset and hospitalization over satisfaction with job, if replicated, may inform and improve strategies for the promotion of health and wellbeing. That satisfaction with marriage predicted lower odds of chronic condition health onset and future hospitalization is in line with research that mainly drew western samples [13], suggesting the generalizability of the role of satisfaction with marriage on physical health across two distinct cultures (i.e., collectivistic vs individualistic).

## Limitations

The findings of the present study need to be interpreted in light of several limitations. First, self-report measures were used to assess indicators of physical health, which may bias the study findings. However, this concern was slightly alleviated in the case of chronic health condition, because the wording of this question reminded participants to only report doctor-diagnosed conditions. Future studies that retrieve information that is not self-reported such as data on mortality of the studied population are urgently needed. Second, while multiple items were used to assess satisfaction with marriage and job, some scales including perceived general health and satisfaction with medical services were assessed using single-item questions. The use of single-item questions likely fails to fully capture the constructs of interest. Future researchers are encouraged to apply more comprehensive measures of life satisfaction domains. Likewise, the number of domains was limited by the design of the CFPS panel study. Future cohort studies are encouraged to incorporate questions that tap into more domains of life satisfaction so that more domains could be compared with each other, offering a more complete picture of mechanisms driving the consistently identified link between overall life satisfaction and physical health across cultures. In addition, another limitation of the study is that there remains the possibility of reverse causality despite the use of longitudinal design and the incorporation of theoretically important covariates. While we control for a wide range of covariates, because of the nature of the study design, strong causal inferences could not be made. Finally, the follow-up period was relatively short, in part because the domains were not consistently measured. Future studies with longer observation time will further shed light on the links between specific domains of life satisfaction and physical health.

## Conclusions

Extending studies on life satisfaction and health, we examined a large Chinese adult cohort and found that both levels and changes in three life satisfaction domains were prospectively associated with perceived physical health. Among these domains, satisfaction with marriage was associated with all indicators of physical health and might serve as an ideal target for novel interventions aimed at improving physical health. 

## Supplementary Information

**Additional file 1:** **Table S1. **Patterns of Assessments and Number of Scale Items for Each Domain of Life Satisfaction Across Waves. **Table S2. **Multivariable Linear Regression Model Predicting Perceived Physical Additionally Adjusted for Big-Five Personality Traits. **Table S3. **Multivariable Logistic Regression Model Predicting Chronic Health Condition Onset Additionally Adjusted for Big-Five Personality Traits. **Table S4. **Multivariable Logistic Regression Model Predicting Hospitalization Additionally Adjusted for Big-Five Personality Traits. **Table S5. **Multivariable Linear Regression Model Predicting Perceived Physical Using Single-Item Questions. **Table S6. **Multivariable Logistic Regression Model Predicting Chronic Health Condition Onset Using Single-Item Questions. **Table S7. **Multivariable Logistic Regression Model Predicting Hospitalization Using Single-Item Questions. **Table S8. **Multivariable Linear Regression Model Predicting Perceived Physical Health Using Ten Imputed Datasets. **Table S9. **Multivariable Logistic Regression Model Predicting Chronic Health Condition Onset Using Ten Imputed Datasets. **Table S10. **Multivariable Logistic Regression Model Predicting Hospitalization Using Ten Imputed Datasets. **Table S11. **Multivariable Linear Regression Model Predicting Perceived Physical Health Using Wave 2014 and 2020. **Table S12. **Multivariable Logistic Regression Model Predicting Chronic Health Condition Onset Using Wave 2014 and 2020. **Table S13. **Multivariable Logistic Regression Model Predicting Hospitalization Using Wave 2014 and 2020. **Table S14. **Multivariable Linear Regression Model Predicting Perceived Physical Health Using Wave 2014 and 2018. **Table S15. **Multivariable Logistic Regression Model Predicting Chronic Health Condition Onset Using Wave 2014 and 2018. **Table S16. **Multivariable Logistic Regression Model Predicting Hospitalization Using Wave 2014 and 2018.

## Data Availability

The datasets used and/or analyzed during the current study available from the corresponding author on reasonable request.
