# Supplementary material for: Domains of life satisfaction and perceived health and incidence of chronic illnesses and hospitalization: evidence from a large population-based Chinese cohort
Source: BMC Public Health. 2022 Sep 8;22:1703. doi: 10.1186/s12889-022-14119-3 (PMC9454222; doi:10.1186/s12889-022-14119-3)
Supplement: Supplementary file 1 — Additional file 1: Table S1. Patterns of Assessments and Number of Scale Items for Each Domain of Life Satisfaction Across Waves. Table S2. Multivariable Linear Regression Model Predicting Perceived Physical Additionally Adjusted for Big-Five Personality Traits. Table S3. Multivariable Logistic Regression Model Predicting Chronic Health Condition Onset Additionally Adjusted for Big-Five Personality Traits. Table S4. Multivariable Logistic Regression Model Predicting Hospitalization Additionally Adjusted for Big-Five Personality Traits. Table S5. Multivariable Linear Regression Model Predicting Perceived Physical Using Single-Item Questions. Table S6. Multivariable Logistic Regression Model Predicting Chronic Health Condition Onset Using Single-Item Questions. Table S7. Multivariable Logistic Regression Model Predicting Hospitalization Using Single-Item Questions. Table S8. Multivariable Linear Regression Model Predicting Perceived Physical Health Using Ten Imputed Datasets. Table S9. Multivariable Logistic Regression Model Predicting Chronic Health Condition Onset Using Ten Imputed Datasets. Table S10. Multivariable Logistic Regression Model Predicting Hospitalization Using Ten Imputed Datasets. Table S11. Multivariable Linear Regression Model Predicting Perceived Physical Health Using Wave 2014 and 2020. Table S12. Multivariable Logistic Regression Model Predicting Chronic Health Condition Onset Using Wave 2014 and 2020. Table S13. Multivariable Logistic Regression Model Predicting Hospitalization Using Wave 2014 and 2020. Table S14. Multivariable Linear Regression Model Predicting Perceived Physical Health Using Wave 2014 and 2018. Table S15. Multivariable Logistic Regression Model Predicting Chronic Health Condition Onset Using Wave 2014 and 2018. Table S16. Multivariable Logistic Regression Model Predicting Hospitalization Using Wave 2014 and 2018. [file 12889_2022_14119_MOESM1_ESM.docx]

**Supplemental Material**

**Table S1.** Patterns of Assessments and Number of Scale Items for Each Domain of Life Satisfaction Across Waves

**Table S2.** Multivariable Linear Regression Model Predicting Perceived Physical Additionally Adjusted for Big-Five Personality Traits
**Table S3.** Multivariable Logistic Regression Model Predicting Chronic Health Condition Onset Additionally Adjusted for Big-Five Personality Traits
**Table S4.** Multivariable Logistic Regression Model Predicting Hospitalization Additionally Adjusted for Big-Five Personality Traits

**Table S5.** Multivariable Linear Regression Model Predicting Perceived Physical Using Single-Item Questions
**Table S6.** Multivariable Logistic Regression Model Predicting Chronic Health Condition Onset Using Single-Item Questions
**Table S7.** Multivariable Logistic Regression Model Predicting Hospitalization Using Single-Item Questions
**Table S8.** Multivariable Linear Regression Model Predicting Perceived Physical Health Using Ten Imputed Datasets
**Table S9.** Multivariable Logistic Regression Model Predicting Chronic Health Condition Onset Using Ten Imputed Datasets
**Table S10.** Multivariable Logistic Regression Model Predicting Hospitalization Using Ten Imputed Datasets
**Table S11.** Multivariable Linear Regression Model Predicting Perceived Physical Health Using Wave 2014 and 2020
**Table S12.** Multivariable Logistic Regression Model Predicting Chronic Health Condition Onset Using Wave 2014 and 2020
**Table S13.** Multivariable Logistic Regression Model Predicting Hospitalization Using Wave 2014 and 2020

**Table S14.** Multivariable Linear Regression Model Predicting Perceived Physical Health Using Wave 2014 and 2018
**Table S15.** Multivariable Logistic Regression Model Predicting Chronic Health Condition Onset Using Wave 2014 and 2018
**Table S16.** Multivariable Logistic Regression Model Predicting Hospitalization Using Wave 2014 and 2018

**Table S.1** Patterns of Assessments and Number of Scale Items for Each Domain of Life Satisfaction Across Waves

| Domain of life satisfaction | 2010 | Item Count 2010 | 2012 | Item Count 2012 | 2014 | Item Count 2014 | 2016 | Item Count 2016 | 2018 | Item Count 2018 | 2020 | Item Count 2020 |
| --- | --- | --- | --- | --- | --- | --- | --- | --- | --- | --- | --- | --- |
| Marriage | No | – | No | – | Yes | 3 | No | – | Yes | 3 | Yes | 4 |
| Job | Yes | 6 | No | – | Yes | 2 | Yes | 6 | Yes | 6 | Yes | 6 |
| Medical service | Yes | 1 | Yes | 1 | Yes | 1 | Yes | 1 | Yes | 1 | Yes | 1 |
|  |  |  |  |  |  |  |  |  |  |  |  |  |

*Note*. An additional item was used to tap into satisfaction with marriage only in 2020, and therefore was not used across waves. “Yes” denotes that a specific domain was assessed in the corresponding wave, while “No” denotes otherwise. – denotes not available.

**Table S.2** Multivariable Linear Regression Model Predicting Perceived Physical Additionally Adjusted for Big-Five Personality Traits

| Exposure variable | Perceived Physical Health | | | |
| --- | --- | --- | --- | --- |
|  | *β* | *t* | 95% CI | *P* |
| Job Satisfaction Level | 0.11 | 8.99 | 0.09 to 0.13 | <.0001 |
| Marrital Satisfaction Level | 0.08 | 7.17 | 0.06 to 0.10 | <.0001 |
| Medical Service Satisfaction Level | 0.04 | 3.16 | 0.01 to 0.06 | <.01 |
| Job Satisfaction Change | 0.13 | 10.90 | 0.11 to 0.15 | <.0001 |
| Marrital Satisfaction Change | 0.07 | 6.19 | 0.05 to 0.09 | <.0001 |
| Medical Service Satisfaction Change | 0.04 | 3.25 | 0.02 to 0.07 | <.01 |

*Note*. Model was adjusted for demographics including sex, age, education level, residential possession, family income, and party membership, perceived physical health in baseline wave and additionally adjusted for Big-Five personality traits. CI = confidence interval.

**Table S.3** Multivariable Logistic Regression Model Predicting Chronic Health Condition Onset Additionally Adjusted for Big-Five Personality Traits

| Exposure variable | Chronic Health Condition Onset | | | |
| --- | --- | --- | --- | --- |
|  | OR | *z* | 95% CI | *p* |
| Job Satisfaction Level | 0.95 | -1.10 | 0.86 to 1.04 | .27 |
| Marrital Satisfaction Level | 0.83 | -3.90 | 0.76 to 0.91 | <.0001 |
| Medical Service Satisfaction Level | 0.93 | -1.41 | 0.83 to 1.03 | .16 |
| Job Satisfaction Change | 0.95 | -1.22 | 0.87 to 1.03 | .22 |
| Marrital Satisfaction Change | 0.91 | -2.25 | 0.83 to 0.99 | <.05 |
| Medical Service Satisfaction Change | 0.99 | -0.24 | 0.89 to 1.10 | .81 |

*Note*. Model was adjusted for demographics including sex, age, education level, residential possession, family income, and party membership in the baseline wave and additionally adjusted for Big-Five personality traits. OR = odds ratio. CI = confidence interval.

**Table S.4** Multivariable Logistic Regression Model Predicting Hospitalization Additionally Adjusted for Big-Five Personality Traits

| Exposure variable | Hospitalization | | | |
| --- | --- | --- | --- | --- |
|  | OR | *z* | 95% CI | *p* |
| Job Satisfaction Level | 0.89 | -1.99 | 0.80 to 1.00 | <.05 |
| Marrital Satisfaction Level | 0.87 | -2.70 | 0.78 to 0.96 | <.01 |
| Medical Service Satisfaction Level | 0.99 | -0.15 | 0.88 to 1.11 | .88 |
| Job Satisfaction Change | 0.91 | -1.88 | 0.82 to 1.00 | .06 |
| Marrital Satisfaction Change | 0.91 | -1.95 | 0.83 to 1.00 | .05 |
| Medical Service Satisfaction Change | 0.94 | -1.00 | 0.84 to 1.06 | .32 |

*Note*. Model was adjusted for demographics including sex, age, education level, residential possession, family income, and party membership in the baseline wave and additionally adjusted for Big-Five personality traits. OR = odds ratio. CI = confidence interval.

**Table S.5** Multivariable Linear Regression Model Predicting Perceived Physical Using Single-Item Questions

| Exposure variable | Perceived Physical Health | | | |
| --- | --- | --- | --- | --- |
|  | *β* | *t* | 95% CI | *p* |
| Job Satisfaction Level | 0.11 | 9.18 | 0.09 to 0.14 | <.0001 |
| Marrital Satisfaction Level | 0.06 | 5.70 | 0.04 to 0.09 | <.0001 |
| Medical Service Satisfaction Level | 0.05 | 3.84 | 0.02 to 0.07 | <.001 |
| Job Satisfaction Change | 0.12 | 9.90 | 0.10 to 0.14 | <.0001 |
| Marrital Satisfaction Change | 0.05 | 4.90 | 0.03 to 0.07 | <.0001 |
| Medical Service Satisfaction Change | 0.05 | 4.14 | 0.03 to 0.08 | <.0001 |

*Note*. Model was adjusted for demographics including sex, age, education level, residential possession, family income, and party membership, perceived physical health in baseline wave and additionally adjusted for Big-Five personality traits. CI = confidence interval.

**Table S.6** Multivariable Logistic Regression Model Predicting Chronic Health Condition Onset Using Single-Item Questions

| Exposure variable | Chronic Health Condition Onset | | | |
| --- | --- | --- | --- | --- |
|  | OR | *z* | 95% CI | *p* |
| Job Satisfaction Level | 0.95 | -1.06 | 0.86 to 1.05 | .29 |
| Marrital Satisfaction Level | 0.83 | -4.05 | 0.76 to 0.91 | <.0001 |
| Medical Service Satisfaction Level | 0.92 | -1.49 | 0.83 to 1.02 | .14 |
| Job Satisfaction Change | 0.95 | -1.03 | 0.87 to 1.04 | .30 |
| Marrital Satisfaction Change | 0.90 | -2.44 | 0.83 to 0.98 | <.05 |
| Medical Service Satisfaction Change | 0.97 | -0.49 | 0.88 to 1.08 | .62 |

*Note*. Model was adjusted for demographics including sex, age, education level, residential possession, family income, and party membership in the baseline wave and additionally adjusted for Big-Five personality traits. OR = odds ratio. CI = confidence interval.

**Table S.7** Multivariable Logistic Regression Model Predicting Hospitalization Using Single-Item Questions

| Exposure variable | Hospitalization | | | |
| --- | --- | --- | --- | --- |
|  | OR | *z* | 95% CI | *p* |
| Job Satisfaction Level | 0.91 | -1.75 | 0.81 to 1.01 | .08 |
| Marrital Satisfaction Level | 0.87 | -2.74 | 0.79 to 0.96 | <.01 |
| Medical Service Satisfaction Level | 0.99 | -0.25 | 0.88 to 1.11 | .80 |
| Job Satisfaction Change | 0.90 | -1.90 | 0.81 to 1.00 | .06 |
| Marrital Satisfaction Change | 0.95 | -0.97 | 0.86 to 1.05 | .33 |
| Medical Service Satisfaction Change | 0.94 | -1.14 | 0.83 to 1.05 | .25 |

*Note*. Model was adjusted for demographics including sex, age, education level, residential possession, family income, and party membership in the baseline wave and additionally adjusted for Big-Five personality traits. OR = odds ratio. CI = confidence interval.

**Table S.8** Multivariable Linear Regression Model Predicting Perceived Physical Health Using Ten Imputed Datasets

| Exposure variable | Perceived Physical Health | | | |
| --- | --- | --- | --- | --- |
|  | *β* | *t* | 95% CI | *p* |
| Job Satisfaction Level | 0.12 | 9.80 | 0.09 to 0.14 | <.0001 |
| Marrital Satisfaction Level | 0.08 | 7.84 | 0.06 to 0.10 | <.0001 |
| Medical Service Satisfaction Level | 0.04 | 3.03 | 0.01 to 0.06 | <.01 |
| Job Satisfaction Change | 0.13 | 11.01 | 0.11 to 0.15 | <.0001 |
| Marrital Satisfaction Change | 0.07 | 6.81 | 0.05 to 0.09 | <.0001 |
| Medical Service Satisfaction Change | 0.04 | 3.58 | 0.02 to 0.07 | <.001 |

*Note*. Model was adjusted for demographics including sex, age, education level, residential possession, family income, and party membership, and perceived physical health in the baseline wave. CI = confidence interval.

**Table S.9** Multivariable Logistic Regression Model Predicting Chronic Health Condition Onset Using Ten Imputed Datasets

| Exposure variable | Chronic Health Condition Onset | | | |
| --- | --- | --- | --- | --- |
|  | OR | *z* | 95% CI | *p* |
| Job Satisfaction Level | 0.93 | -1.42 | 0.85 to 1.03 | .16 |
| Marrital Satisfaction Level | 0.85 | -3.50 | 0.78 to 0.93 | <.001 |
| Medical Service Satisfaction Level | 0.92 | -1.57 | 0.83 to 1.02 | .12 |
| Job Satisfaction Change | 0.95 | -1.27 | 0.87 to 1.03 | .21 |
| Marrital Satisfaction Change | 0.92 | -2.10 | 0.84 to 0.99 | <.05 |
| Medical Service Satisfaction Change | 0.97 | -0.60 | 0.88 to 1.07 | .55 |

*Note*. Model was adjusted for demographics including sex, age, education level, residential possession, family income, and party membership in the baseline wave. OR = odds ratio. CI = confidence interval.

**Table S.10** Multivariable Logistic Regression Model Predicting Hospitalization Using Ten Imputed Datasets

| Exposure variable | Hospitalization | | | |
| --- | --- | --- | --- | --- |
|  | OR | *z* | 95% CI | *p* |
| Job Satisfaction Level | 0.90 | -2.04 | 0.80 to 1.00 | <.05 |
| Marrital Satisfaction Level | 0.88 | -2.62 | 0.79 to 0.97 | <.01 |
| Medical Service Satisfaction Level | 1.00 | -0.08 | 0.89 to 1.12 | .94 |
| Job Satisfaction Change | 0.91 | -1.82 | 0.82 to 1.01 | .07 |
| Marrital Satisfaction Change | 0.92 | -1.81 | 0.84 to 1.01 | .07 |
| Medical Service Satisfaction Change | 0.96 | -0.67 | 0.86 to 1.08 | .50 |

*Note*. Model was adjusted for demographics including sex, age, education level, residential possession, family income, and party membership in the baseline wave. OR = odds ratio. CI = confidence interval.

**Table S.11** Multivariable Linear Regression Model Predicting Perceived Physical Health Using Wave 2014 and 2020

| Exposure variable | Perceived Physical Health | | | |
| --- | --- | --- | --- | --- |
|  | *β* | *t* | 95% CI | *p* |
| Job Satisfaction Level | 0.14 | 10.31 | 0.11 to 0.17 | <.0001 |
| Marrital Satisfaction Level | 0.08 | 6.07 | 0.05 to 0.10 | <.0001 |
| Medical Service Satisfaction Level | 0.05 | 3.84 | 0.03 to 0.08 | <.001 |
| Job Satisfaction Change | 0.16 | 11.30 | 0.13 to 0.18 | <.0001 |
| Marrital Satisfaction Change | 0.10 | 8.52 | 0.08 to 0.13 | <.0001 |
| Medical Service Satisfaction Change | 0.07 | 5.39 | 0.05 to 0.10 | <.0001 |

*Note*. Model was adjusted for demographics including sex, age, education level, residential possession, family income, party membership, and perceived physical health in the baseline wave (2014). CI = confidence interval.

**Table S.12** Multivariable Logistic Regression Model Predicting Chronic Health Condition Onset Using Wave 2014 and 2020

| Exposure variable | Chronic Health Condition Onset | | | |
| --- | --- | --- | --- | --- |
|  | OR | *z* | 95% CI | *p* |
| Job Satisfaction Level | 0.92 | -1.77 | 0.83 to 1.01 | .08 |
| Marrital Satisfaction Level | 0.85 | -3.52 | 0.78 to 0.93 | <.001 |
| Medical Service Satisfaction Level | 0.97 | -0.55 | 0.89 to 1.07 | .59 |
| Job Satisfaction Change | 0.92 | -1.70 | 0.84 to 1.01 | .09 |
| Marrital Satisfaction Change | 0.87 | -3.13 | 0.80 to 0.95 | <.05 |
| Medical Service Satisfaction Change | 0.97 | -0.62 | 0.89 to 1.06 | .54 |

*Note*. Model was adjusted for demographics including sex, age, education level, residential possession, family income, and party membership in the baseline wave (2014). OR = odds ratio. CI = confidence interval.

**Table S.13** Multivariable Logistic Regression Model Predicting Hospitalization Using Wave 2014 and 2020

| Exposure variable | Hospitalization | | | |  |
| --- | --- | --- | --- | --- | --- |
|  | OR | *z* | 95% CI | *p* | |
| Job Satisfaction Level | 0.86 | -2.65 | 0.77 to 0.96 | <.01 | |
| Marrital Satisfaction Level | 0.90 | -1.90 | 0.82 to 1.00 | .06 | |
| Medical Service Satisfaction Level | 1.03 | 0.54 | 0.92 to 1.15 | .59 | |
| Job Satisfaction Change | 0.91 | -1.76 | 0.82 to 1.01 | .08 | |
| Marrital Satisfaction Change | 0.88 | -2.59 | 0.80 to 0.97 | <.01 | |
| Medical Service Satisfaction Change | 0.99 | -0.10 | 0.89 to 1.11 | .92 | |

*Note*. Model was adjusted for demographics including sex, age, education level, residential possession, family income, and party membership in the baseline wave. OR = odds ratio. CI = confidence interval.

**Table S.14** Multivariable Linear Regression Model Predicting Perceived Physical Health Using Wave 2014 and 2018

| Exposure variable | Perceived Physical Health | | | |
| --- | --- | --- | --- | --- |
|  | *β* | *t* | 95% CI | *p* |
| Job Satisfaction Level | 0.11 | 8.97 | 0.08 to 0.13 | <.0001 |
| Marrital Satisfaction Level | 0.07 | 6.72 | 0.05 to 0.09 | <.0001 |
| Medical Service Satisfaction Level | 0.06 | 5.83 | 0.04 to 0.09 | <.0001 |
| Job Satisfaction Change | 0.11 | 9.25 | 0.08 to 0.13 | <.0001 |
| Marrital Satisfaction Change | 0.08 | 7.72 | 0.06 to 0.11 | <.0001 |
| Medical Service Satisfaction Change | 0.08 | 7.63 | 0.06 to 0.11 | <.0001 |

*Note*. Model was adjusted for demographics including sex, age, education level, residential possession, family income, party membership, and perceived physical health in the baseline wave (2014). CI = confidence interval.

**Table S.15** Multivariable Logistic Regression Model Predicting Chronic Health Condition Onset Using Wave 2014 and 2018

| Exposure variable | Chronic Health Condition Onset | | | |
| --- | --- | --- | --- | --- |
|  | OR | *z* | 95% CI | *p* |
| Job Satisfaction Level | 0.95 | -1.13 | 0.88 to 1.03 | .26 |
| Marrital Satisfaction Level | 0.94 | -1.51 | 0.87 to 1.02 | .13 |
| Medical Service Satisfaction Level | 0.90 | -2.49 | 0.83 to 0.98 | < .05 |
| Job Satisfaction Change | 0.94 | -1.58 | 0.87 to 1.02 | .11 |
| Marrital Satisfaction Change | 0.93 | -1.79 | 0.87 to 1.01 | .07 |
| Medical Service Satisfaction Change | 0.96 | -1.08 | 0.89 to 1.04 | .28 |

*Note*. Model was adjusted for demographics including sex, age, education level, residential possession, family income, and party membership in the baseline wave (2014). OR = odds ratio. CI = confidence interval.

**Table S.16** Multivariable Logistic Regression Model Predicting Hospitalization Using Wave 2014 and 2018

| Exposure variable | Hospitalization | | | |
| --- | --- | --- | --- | --- |
|  | OR | *z* | 95% CI | *p* |
| Job Satisfaction Level | 0.99 | -0.12 | 0.91 to 1.08 | 0.90 |
| Marrital Satisfaction Level | 0.89 | -2.68 | 0.82 to 0.97 | <.01 |
| Medical Service Satisfaction Level | 1.09 | 1.92 | 1.00 to 1.18 | 0.05 |
| Job Satisfaction Change | 1.01 | 0.25 | 0.93 to 1.10 | 0.80 |
| Marrital Satisfaction Change | 0.96 | -1.02 | 0.89 to 1.04 | 0.31 |
| Medical Service Satisfaction Change | 1.08 | 1.87 | 1.00 to 1.17 | 0.06 |

*Note*. Model was adjusted for demographics including sex, age, education level, residential possession, family income, and party membership in the baseline wave. OR = odds ratio. CI = confidence interval.
